# A Potentiometric Sensor Based on a Molecularly Imprinted Polymer Modified with Multi-Walled Carbon Nanotubes for the Selective Determination of Thiabendazole Residues in Fruit Peels

**DOI:** 10.3390/s26206474

**Published:** 2026-10-13

**Authors:** Mirela Samardžić, Andrea Dandić, Izabella Doris Panić, Mateja Budetić

**Affiliations:** Department of Chemistry, Josip Juraj Strossmayer University of Osijek, Cara Hadrijana 8/A, 31000 Osijek, Croatia; mirelas@kemija.unios.hr (M.S.); andreajuric@kemija.unios.hr (A.D.);

**Keywords:** molecularly imprinted polymer, potentiometric sensor, ion-selective electrode, thiabendazole, pesticide residues

## Abstract

**Highlights:**

- A novel potentiometric sensor integrating molecularly imprinted polymers with multi-walled carbon nanotubes.
- A fast response and a wide linear range for thiabendazole determination.
- Prepared orange, lemon, and banana peel extracts for thiabendazole determination.

**Abstract:**

Thiabendazole (TBZ) is a widely used post-harvest fungicide for fruit protection. Therefore, a new potentiometric sensor for TBZ determination based on a molecularly imprinted polymer (MIP) modified with multi-walled carbon nanotubes (MWCNTs) was developed. Different plasticizers, amounts of sensor material, and MWCNTs were tested during sensor optimization. The best sensor response was obtained for the membrane containing dibutyl phthalate (DBP) as a plasticizer, 3 wt% sensor material, and 2 wt% MWCNTs. The selected sensor showed a slope of 59.7 mV/decade of activity, a limit of detection (LOD) of 3.2·10^−7^ M, and a wide measuring range (6.2·10^−7^–1.0·10^−3^ M). The response time was 4 s, with a stable signal and a potential drift of 0.72 mV/h. The sensor was applied for TBZ determination in standard solutions and prepared fruit-peel extracts (orange, banana, and lemon).

## 1. Introduction

A pesticide is any substance used to eliminate, repel, or control organisms of animal or plant origin that are considered harmful or undesirable in agricultural and domestic environments [1]. Modern agriculture relies heavily on the use of pesticides and fungicides to ensure stable yields and reduce losses caused by plant diseases. Although this approach has clear economic and agronomic advantages, it also raises several issues related to food safety and environmental impact [2]. The continuously increasing use of pesticides has contaminated the environment, harmed agriculture, and put human health at risk [2,3,4]. As a result, strict regulations have been developed and implemented to monitor the use of pesticides and their residues in different parts of the environment [4,5,6]. Pesticides include several groups of compounds, including herbicides used to control weeds and other unwanted vegetation, insecticides designed to manage a broad range of insect pests, and fungicides, among which thiabendazole (TBZ) is widely recognized. TBZ also belongs to the benzimidazole class of fungicides and is extensively used, particularly for the postharvest protection of fruits [7]. Its high efficacy against fungal pathogens has contributed to its widespread application; however, concerns have been raised regarding the potential adverse effects associated with prolonged exposure. The presence of amine and thiazole functional groups in the TBZ molecule contributes to its ability to interact with biological targets, including cellular proteins, and is associated with its antifungal activity through interference with essential cellular processes. While these structural features may influence its physicochemical behavior and mobility in plant systems, TBZ uptake and translocation depend on multiple environmental and biological factors [8,9]. However, the main limitation of TBZ is its very low water solubility, which restricts its removal by simple washing after postharvest application. As a result, it may persist on treated commodities and potentially enter aquatic environments and beverages through agricultural runoff and food processing pathways [10,11]. Consequently, its extensive use has raised concerns regarding potential adverse effects on human health, including hepatotoxicity, thyroid hormone disruption, anemia, and carcinogenic risk [12]. Considering global regulatory frameworks, monitoring and control of TBZ residues in food are of critical importance. The determination of its concentration in various matrices enables compliance verification with established maximum residue limits (MRLs) in different countries and represents a key component of consumer health protection [13].

TBZ is most commonly determined using chromatographic techniques such as high-performance liquid chromatography (HPLC) and gas chromatography-mass spectrometry (GC-MS) [14,15,16,17]. Although these methods provide high sensitivity and accuracy, they require complex and time-consuming sample preparation procedures, involve the use of large volumes of organic solvents, and entail high operational costs. Furthermore, they rely on expensive instrumentation that requires skilled personnel as well as regular maintenance and calibration [18,19,20]. Given these limitations of the most widely used methods for TBZ determination, electrochemical sensors represent a suitable alternative. Among them, potentiometric sensors offer several advantages, including rapid analysis, simple instrumentation, minimal sample preparation, and reduced reagent consumption, also making them attractive from a green chemistry perspective [21,22]. The applicability of potentiometric sensors largely depends on their selectivity, which has driven the development of various functional materials capable of specific recognition of the target analyte [23,24,25]. This field has experienced rapid progress in recent years, with an increasing emphasis on the use of nanomaterials in sensor fabrication [26,27].

Ion-selective electrodes (ISEs) are among the most widely used potentiometric sensors and have long been employed for the determination of inorganic and organic ions in complex samples. Their performance relies on an ion-selective membrane, typically composed of a polymer matrix, a plasticizer, and a sensing material responsible for the selective recognition of the target analyte [28,29,30]. Recent research has focused on improving the selectivity of ISEs through the development of novel functional sensing materials, particularly molecularly imprinted polymers (MIPs). These synthetic materials contain specific binding sites complementary in size, shape, and functional group arrangement to the target molecule, enabling highly selective analyte recognition. Owing to their high chemical stability and resistance to environmental changes, MIPs represent attractive alternatives to biological receptors for sensor applications [31,32,33].

Although MIPs provide high selectivity toward target molecules, their performance may be limited by restricted mass transfer and slow analyte diffusion within the polymer matrix [31]. To overcome these limitations, MIPs are often combined with nanostructured carbon materials, such as carbon nanotubes and graphene [34,35,36,37]. Owing to their high specific surface area and surface-modification possibilities, these materials are suitable for integration with polymer-based sensing materials and can contribute to improved sensor performance. Consequently, MIP-based electrodes incorporating carbon nanomaterials represent a promising approach for the development of highly selective and reliable ion-selective sensors [38,39,40].

Based on these findings, this study focused on the development of a potentiometric sensor integrating an MIP with multi-walled carbon nanotubes (MWCNTs) for TBZ determination. In our previous studies, two different sensing approaches were investigated: a potentiometric sensor based on a hybrid sensing material consisting of functionalized MWCNTs and TBZ (MWCNT-OSO_3_^−^TBZ^+^) [23] and an MIP-based potentiometric sensor without the addition of MWCNTs [41]. In the present study, these approaches were further developed by treating MWCNTs with methacrylic acid (MAA) and subsequently incorporating the MAA-treated MWCNTs into the TBZ-imprinted polymer, together with the addition of free MWCNTs to the polyvinyl chloride (PVC) membrane, the amount of which was systematically optimized. The MIP provides selective molecular recognition of TBZ, while the incorporation of MWCNTs was investigated with the aim of improving the overall performance and stability of the potentiometric sensor. To the best of our knowledge, no potentiometric sensor for TBZ determination combining an MWCNT-modified MIP with additional free MWCNTs incorporated into the sensing membrane has previously been reported. Therefore, the novelty of the present work lies primarily in the development and evaluation of this combined sensing design while maintaining simple sensor preparation and favorable analytical performance.

## 2. Materials and Methods

### 2.1. Reagents and Materials

Reagents and solvents for the synthesis of the sensor material: MWCNTs (io-li-tec, Heilbronn, Germany, o.d. 60–100 nm, length 5–15 μm, 95%), trimethylolpropane triacrylate (TRIM, Thermo Fisher Scientific, Waltham, MA, USA), methacrylic acid (MAA, Thermo Fisher Scientific, Waltham, MA, USA), benzoyl peroxide (BZO, Merck, Darmstadt, Germany), TBZ (Thermo Fisher Scientific, Waltham, MA, USA), acetonitrile (ACN, VWR Chemicals, Radnor, PA, USA), methanol (MeOH, Thermo Fisher Scientific, Waltham, MA, USA), acetone (Thermo Fisher Scientific, Waltham, MA, USA), and acetic acid (AcOH, Lab expert d.o.o., Zagreb, Croatia).

Reagents and solvents for membrane preparation: PVC (Fluka, Buchs, Switzerland), dibutyl sebacate (DS, Fluka, Buchs, Switzerland), *o*-nitrophenyl octyl ether (*o*-NPOE, Fluka, Buchs, Switzerland), bis(2-ethylhexyl) sebacate (BEHS, Fluka, Buchs, Switzerland), dibutyl phthalate (DBP, Fluka, Buchs, Switzerland), MWCNTs, and tetrahydrofuran (THF, Thermo Fisher Scientific, Waltham, MA, USA).

Hydrochloric acid (HCl, Carlo Erba, Milan, Italy) and sodium hydroxide (NaOH, T.T.T., Zagreb, Croatia) were used to adjust the pH value of the solution.

TBZ and HCl were used to prepare the analyte solution.

The selectivity of the sensor was tested using prepared solutions of various potentially interfering ionic and organic species: potassium chloride (KCl, GRAM-MOL, Zagreb, Croatia), sodium chloride (NaCl, GRAM-MOL, Zagreb, Croatia), ammonium nitrate (NH_4_NO_3_, Kemika, Zagreb, Croatia), lithium chloride (LiCl, GRAM-MOL, Zagreb, Croatia), copper(II) chloride (CuCl_2_, Kemika, Zagreb, Croatia), calcium nitrate (Ca(NO_3_)_2_, Carlo Erba Reagents, Milan, Italy), magnesium chloride (MgCl_2_, GRAM-MOL, Zagreb, Croatia), zinc nitrate (Zn(NO_3_)_2_, Thermo Fisher Scientific, Waltham, MA, USA), iron(III) chloride (FeCl_3_, Thermo Fisher Scientific, Waltham, MA, USA), iron(II) sulfate (FeSO_4_, Acros Organics, Geel, Belgium), imazalil (Dr. Ehrenstorfer, Augsburg, Germany), fuberidazole (Dr. Ehrenstorfer, Augsburg, Germany), and carbendazim (Dr. Ehrenstorfer, Augsburg, Germany).

All solutions were prepared using deionized water with a conductivity of 0.055 µS/cm.

Lemon, orange, and banana peels purchased from a local store in Osijek were used to determine TBZ in real samples.

### 2.2. Apparatus

FT-IR spectra were recorded using an instrument FTIR 88400S (Shimadzu, Kyoto, Japan) in the range of 4000–400 cm^−1^ (resolution 4 cm^−1^). TGA (Thermogravimetric analysis) and DSC (Differential scanning calorimetry) analyses were performed using a simultaneous TGA-DSC analyzer (Mettler-Toledo TGA/DSC 1, Columbus, OH, USA). The samples were heated in a nitrogen atmosphere (200 mL min^−1^) in 70 μL alumina pans up to 800 °C at a rate of 10 °C min^−1^.

During the synthesis and purification of the sensor material, a centrifuge (A Centric 200R, Intertech d.o.o., Radomlje, Slovenia), a rotary evaporator (Büchi Rotavapor R-300, Büchi Labortechnik AG, Flawil, Switzerland), and a Soxhlet extraction apparatus (LLG, Lenz-Laborglas GmbH & Co. KG, Wertheim, Germany) were used.

TBZ solutions and sensor membranes were prepared using an ultrasonic bath (BANDELIN RK-100, Berlin, Germany).

The potentiometric measurement system was composed of 794 Basic Titrino, 806 Exchange unit, 728 Stirrer, silver/silver chloride reference electrode with KCl (*c* = 3 M) as an inner electrolyte, 826 mobile pH meter, combined pH electrode with LiCl salt in ethanol as an inner electrolyte(all from Metrohm, Herisau, Switzerland), working electrode (Philips electrode body IS-561, Glasblaeserei Moeller, Zurich, Switzerland) with NaCl (*c* = 3 M) as an inner electrolyte and self-made membrane, and in-house software.

### 2.3. Preparation of the Sensor

#### 2.3.1. Preparation of MAA-Treated MWCNTs

MWCNTs (200 mg) were placed into a flask after which an aqueous solution of MAA (16 mL, water:MAA = 1:1) was added. The flask was sealed, and the reaction mixture was stirred and heated at 50 °C for 24 h in an oil bath. After completion of the reaction, the mixture was centrifuged (5000 rpm, 5 min) and washed with water and acetone. MAA-treated MWCNTs (MWCNT-MAA) were then dried at 60 °C for 24 h.

#### 2.3.2. Preparation of Sensor Material

MWCNT-MAA (100 mg) was placed in a flask, followed by the addition of the template molecule TBZ (60 mg, 0.30 mmol), ACN (5 mL), and the functional monomer MAA (103 μL, 1.2 mmol). The mixture was agitated for 2 min using a vortex stirrer. Subsequently, the crosslinker (TRIM, 1836 μL, 6 mmol) was added to the reaction mixture. Finally, the radical initiator BZO (56 mg, 0.18 mmol) was added, after which the reaction mixture was purged with nitrogen for 30 min. The flask was sealed and placed in an oil bath at 60 °C for 24 h. The resulting polymer (unwashed MWCNT-MAA-MIP) was subjected to washing with a solution of MeOH/AcOH, 1:1, in a Soxhlet system for 72 h to extract the template TBZ, yielding the washed MWCNT-MAA-MIP. The prepared washed MWCNT-MAA-MIP sample was dried under vacuum using a rotary evaporator for 5 h and then mechanically ground to produce fine powder, which was used as a sensor material.

The non-imprinted polymer (MWCNT-MAA-NIP) was prepared following the same procedure, except that the template molecule TBZ was not used during the synthesis.

#### 2.3.3. Preparation and Conditioning of the Sensor

Membranes with different compositions were prepared by varying the amount of MWCNT-MAA-MIP composite [1 wt% (0.001814 g); 3 wt% (0.005442 g); 5 wt% (0.00907 g); 7 wt% (0.012698 g); 10 wt% (0.01814 g)], the type and amount of plasticizer [DBP: 64 wt% (111.31 μL); 62.67 wt% (108.99 μL); 61.33 wt% (106.67 μL); 60 wt% (104.35 μL); 58 wt% (100.58 μL); DS: 64 wt% (124.03 μL); *o*-NPOE: 64 wt% (111.63 μL); BEHS: 64 wt% (127.02 μL)]. In this study, the membrane composition was additionally modified by incorporating MWCNTs as a solid functional component at different contents [0.5 wt% (0.000907 g), 1 wt% (0.001814 g), 2 wt% (0.003628 g), and 3 wt% (0.005442 g)]. The MWCNTs, together with the other solid membrane components, were added prior to solvent addition, followed by homogenization to obtain the final membrane mixture. This modification was applied to all sensors while maintaining a constant plasticizer-to-PVC ratio of 2:1.

The membrane components were dissolved in 2 mL of THF using an ultrasonic bath. The obtained membrane mixture was placed into a previously prepared glass ring with a diameter of 24 mm fixed onto a glass plate. After solvent evaporation (24 h), membrane disks with a diameter of 7 mm were cut and incorporated into a commercial Philips electrode body.

The new sensor was stored in deionized water between measurements.

### 2.4. Procedure

All potentiometric measurements were carried out at room temperature using a newly prepared MWCNT-MAA-MIP sensor as the working electrode containing 3 M NaCl as the inner electrolyte.

Measurements were performed under continuous magnetic stirring, with the pH adjusted to 2.6, while no ionic strength adjustment was applied. The pH was also adjusted to 2.6 during the interference measurements with the organic compounds. The total solution volumes used were 20 mL for response measurements and 15 mL for the analysis of real samples.

The potentiometric response of the MWCNT-MAA-MIP sensor was determined by direct potentiometric measurements following incremental additions of TBZ standard solution (*c* = 2.0·10^−3^ M and 5.0·10^−5^ M) into distilled water using in-house-developed software.

The influence of 13 potential interfering compounds on the MWCNT-MAA-MIP sensor response was investigated using the fixed interference method [42]. All interferents were tested at a concentration of 1.0·10^−2^ M, except imazalil and fuberidazole, which were tested at a concentration of 2.5·10^−4^ M, and carbendazim, which was tested at a concentration of 5.0·10^−5^ M due to their limited solubility in aqueous solutions. The TBZ concentration range was adjusted according to the interferent concentration level. For interferents tested at 1.0·10^−2^ M, the TBZ concentration range was 1.0·10^−6^ M to 1.0·10^−3^ M, whereas for interferents tested at lower concentrations (2.5·10^−4^ M and 5.0·10^−5^ M), the TBZ concentration range was from 2.5·10^−8^ M to 2.5·10^−5^ M.

The influence of pH on the potentiometric response was examined by adjusting the solution pH within the range of 2–8 using NaOH and HCl solutions with concentrations of 1.0 M, 1.0·10^−1^ M, and 1.0·10^−2^ M.

For real sample analysis, orange, lemon, and banana peels (*m* = 100 g) were used. The chopped peel samples were placed in glass containers and deionized water was added (*V* = 195 mL, 195 mL, and 175 mL, respectively). The different volumes of deionized water resulted from differences in the volume occupied by 100 g of the respective fruit peels; in each case, water was added until the same total volume level of 300 mL was reached. After adjusting the pH to 3, the samples were left standing for 24 h and subsequently filtered through gauze. Before the measurements, the pH value was checked and, if necessary, readjusted to 3. For the analysis of standard TBZ solutions, 15 mL of each solution was used, followed by ten incremental additions of TBZ standard solution (*c* = 2.0·10^−3^ M), consisting of five 0.5 mL and five 1.0 mL additions. For TBZ determination in the prepared fruit-peel extracts, the same series of incremental additions was applied to 15 mL of each extract. For the fortified measurements, 10 mL of the TBZ standard solution was initially added to 15 mL of each extract, followed by the same series of incremental additions.

Before each daily measurement, the sensor was conditioned in a TBZ solution (*c* = 1.0·10^−4^ M) for 30 min, followed by calibration to verify sensor performance.

## 3. Results

### 3.1. Synthesis and Characterization of MWCNT-MAA-MIP-Modified Material

MWCNTs were incorporated into the sensor material because their high specific surface area and surface-modification possibilities make them suitable for integration with polymer-based sensing materials [43].

The sensor material was synthesized through a two-step reaction process. The first step included reaction of native MWCNTs with MAA to prepare MAA-treated MWCNTs, MWCNT-MAA, with MAA present on the MWCNT surface during subsequent polymerization. This treatment was used to facilitate the integration of MWCNTs into the MIP during polymerization [44]. This reaction was carried out according to the procedure described in the literature [39].

The second step of the synthetic pathway involved the reaction of the MWCNT-MAA with MAA as a functional monomer, TRIM as a cross-linking agent, BZO as the initiator and finally TBZ as a target analyte. The synthetic procedure was carried out according to procedures described in the literature [45]. MAA was selected as the functional monomer due to its favorable properties and strong binding affinity toward a wide range of analytes [40]. TRIM was used as a compatible methacrylate-based cross-linker to form a stable three-dimensional polymer network, while BZO served as a free-radical initiator, with the polymerization carried out under a nitrogen atmosphere to prevent side reactions with oxygen [38]. TBZ was extracted from the MIP structure by washing with a solution of MeOH/AcOH, 1:1.

**Figure 1:**
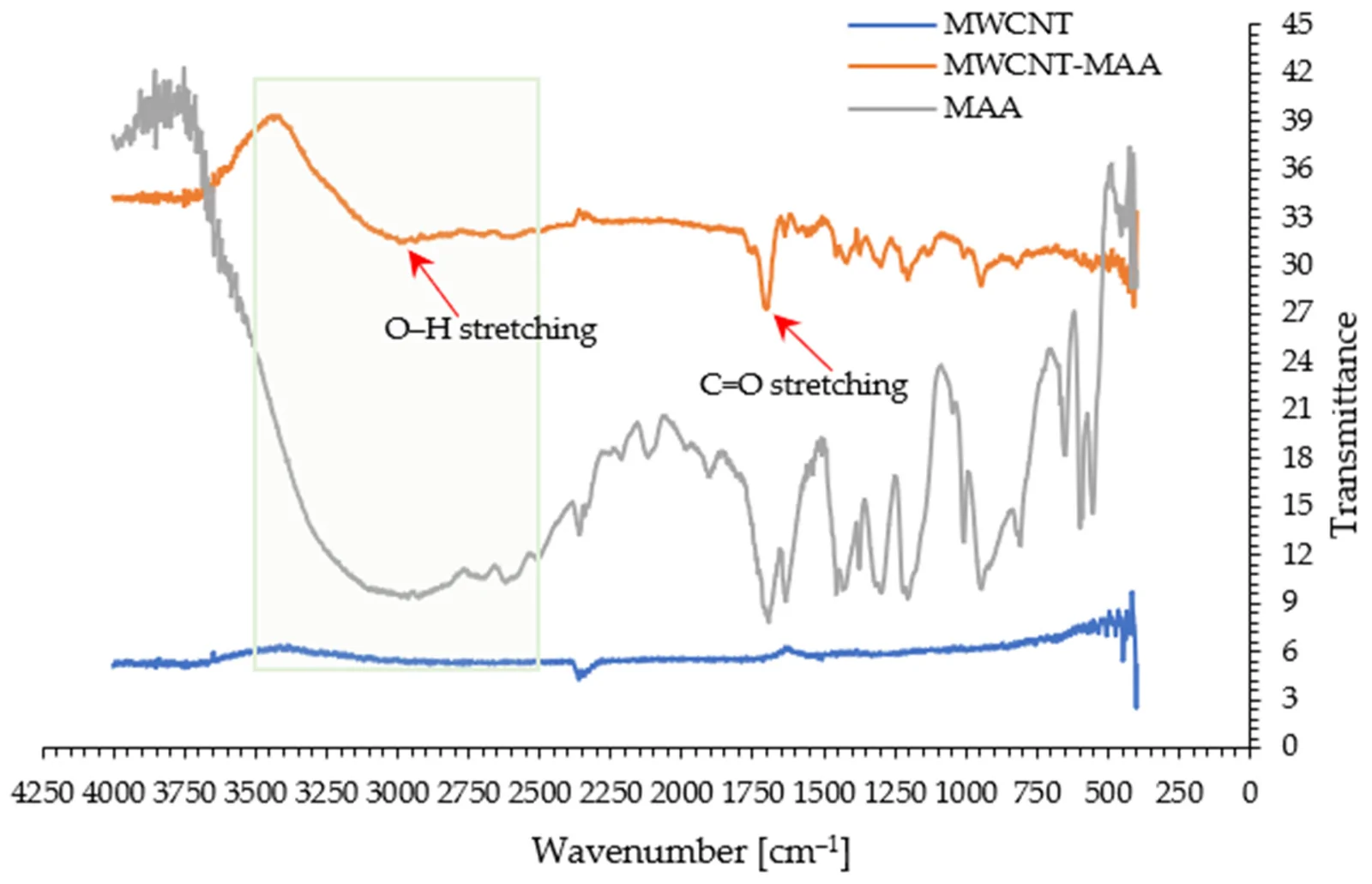
FT-IR spectra of pristine MWCNTs, MAA, and MWCNT-MAA.

The prepared MWCNT-MAA, as well as the washed and unwashed forms of the MIP and the corresponding NIP, were characterized by FT-IR spectroscopy. For the MWCNT-MAA, FT-IR spectra of native MWCNTs, MAA, and MWCNT-MAA were recorded and compared (Figure 1).

The FT-IR spectra of native MWCNTs show no distinct absorption bands, which is typical of their graphitic structure and indicates the absence of surface functional groups. The spectrum of MAA exhibits characteristic bands, including a broad O–H stretching band at 2500–3500 cm^−1^, a strong C=O stretching band around 1700 cm^−1^, and C–O stretching vibrations in the 1300–1000 cm^−1^ region. After modification of MWCNTs with MAA, new bands appear near 1700 cm^−1^ and in the O–H stretching region, indicating changes in the spectral features after modification with MAA.

**Figure 2:**
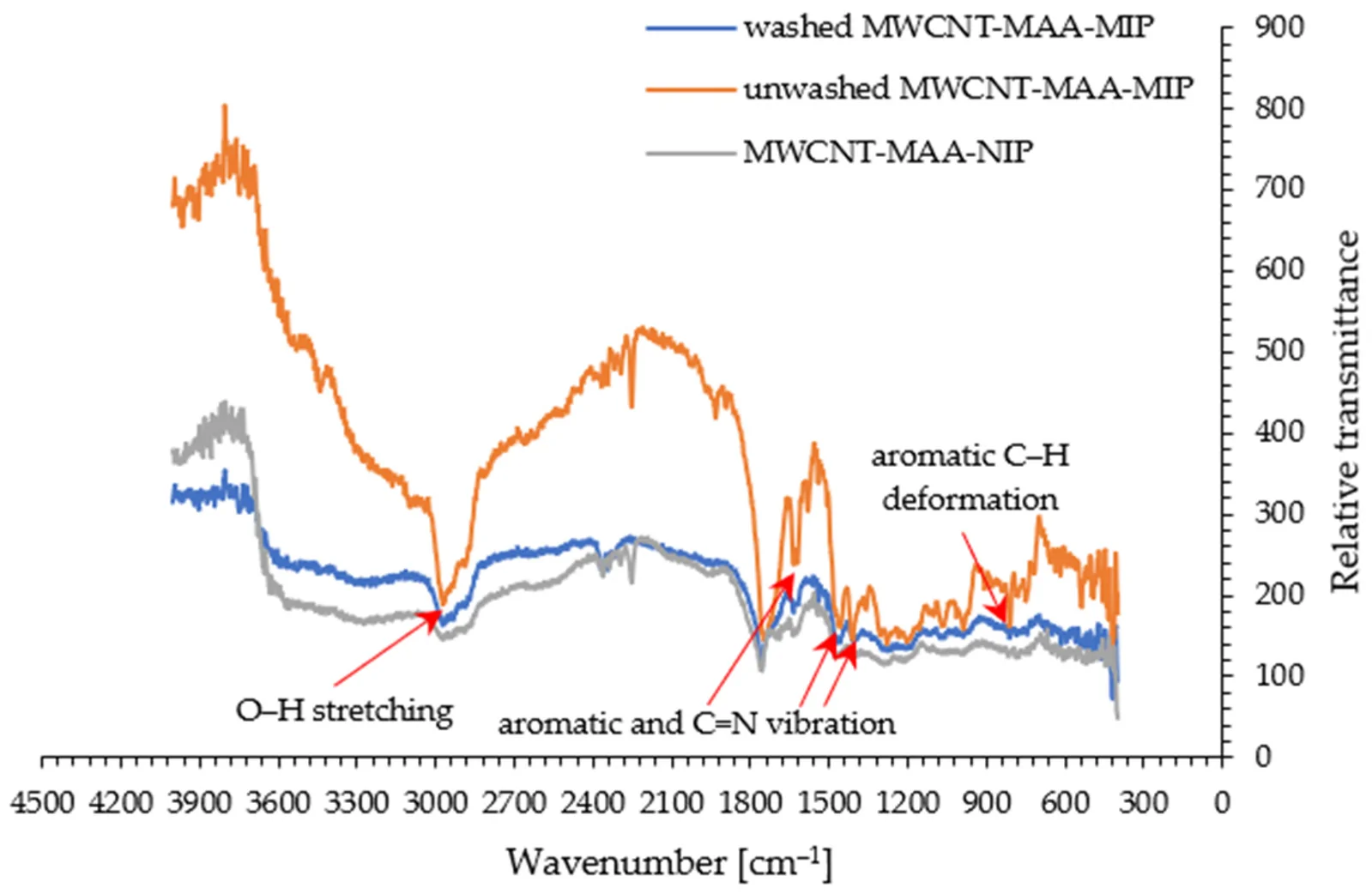
FT-IR spectra of washed MWCNT-MAA-MIP, MWCNT-MAA-NIP, and unwashed MWCNT-MAA-MIP. The spectra were vertically offset for clarity.

**Figure 3:**
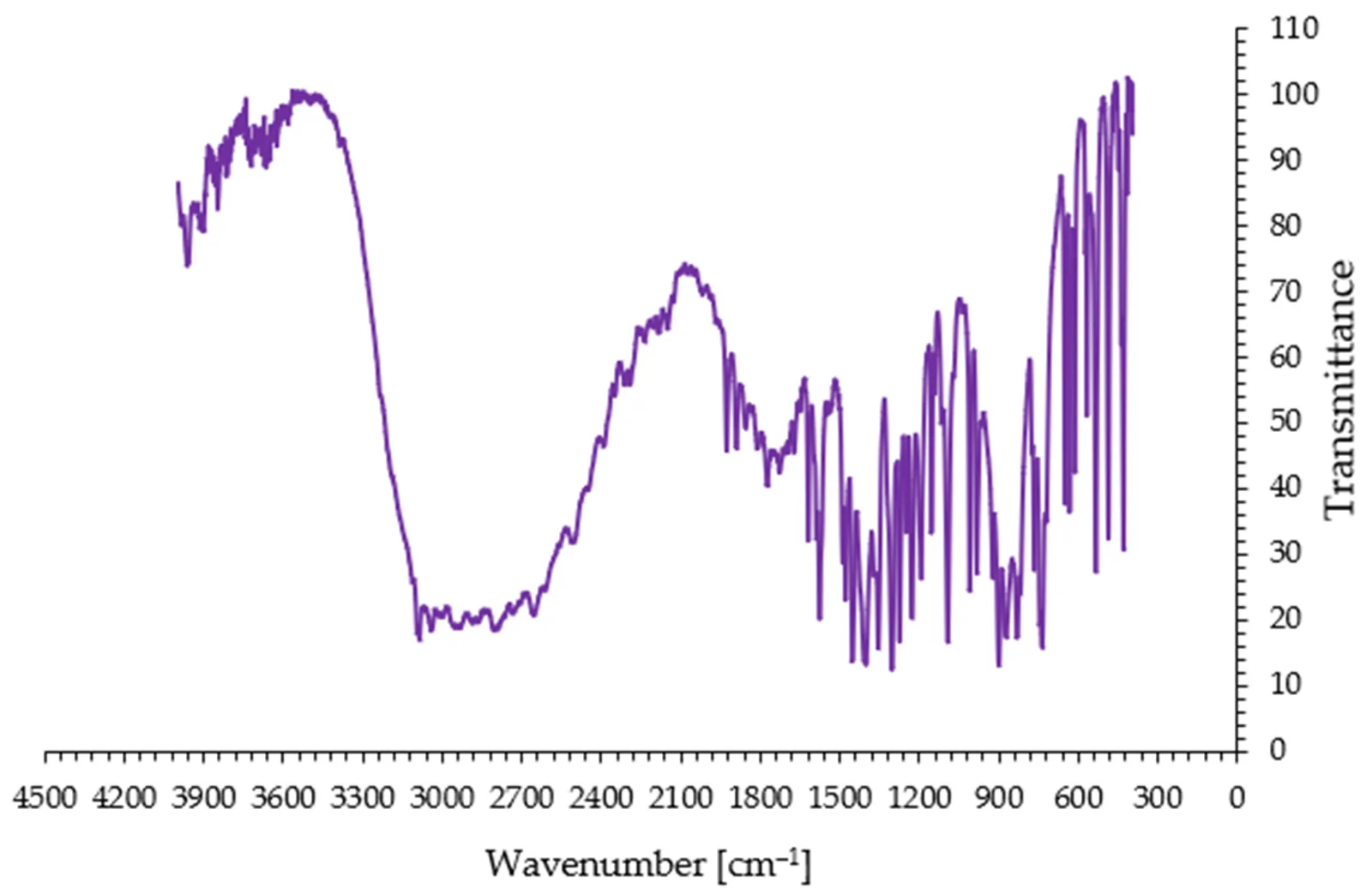
FT-IR spectrum of pure TBZ.

The FT-IR spectra of the MIP, NIP, and washed MIP are presented in Figure 2, while the FT-IR spectrum of pure TBZ is shown in Figure 3. The washed MIP spectrum closely resembles that of the NIP, indicating that the polymer matrix remains intact after the template washing step (Figure 2).

In the IR spectra of all three forms, a band appears near 3000–2900 cm^–1^, which is attributed to the CH_2_ stretching vibrations within the poly(methacrylic) acid (PMAA) framework. Additionally, a peak around 1700 cm^−1^ is present in all forms, suggesting the existence of carbonyl groups derived from the PMAA structure. In our previous work, which involved the preparation of a potentiometric sensor for TBZ determination based on MIP but without functionalization with MWCNT [41], it was highlighted that an important peak in all three forms corresponds to a broad band in the range of 3800–3600 cm^−1^, attributed to O–H stretching in the PMAA structure. In this work, the same observation can be applied. Compared to the FT-IR spectra of washed MWCNT-MAA-MIP and MWCNT-MAA-NIP, where this O–H stretching band is broad, the structure of the unwashed MWCNT-MAA-MIP shows a sharp peak. Since hydrogen bonding is known to broaden O–H [46] stretching bands, the observed difference may reflect changes in the hydrogen bonding environment within the polymeric matrix. The sharper feature observed for the unwashed MIP may therefore be associated with the presence of residual TBZ.

In addition, the FT-IR spectrum of unwashed MWCNT-MAA-MIP displays a weak band at approximately 810 cm^−1^, attributed to the deformation of the aromatic C–H group of TBZ. This assignment is further supported by comparison with the FT-IR spectrum of pure TBZ presented in Figure 3, where a band at a similar wavenumber is observed. This band is absent in the spectra of washed MWCNT-MAA-MIP and MWCNT-MAA-NIP, indicating that TBZ was not detected by FT-IR in these two forms. Ultimately, the unwashed MWCNT-MAA-MIP exhibits stronger absorption bands across the spectrum, corresponding to the presence of the template molecule, TBZ. Aromatic and C=N vibrations (~1500–1600 cm^−1^) are prominent in the unwashed MWCNT-MAA-MIP but absent in the washed MWCNT-MAA-MIP and MWCNT-MAA-NIP, suggesting that TBZ-related bands were no longer detectable after the washing step. These characteristic vibrations are also observed in the FT-IR spectrum of pure TBZ shown in Figure 3, further supporting their assignment to the TBZ template.

**Figure 4:**
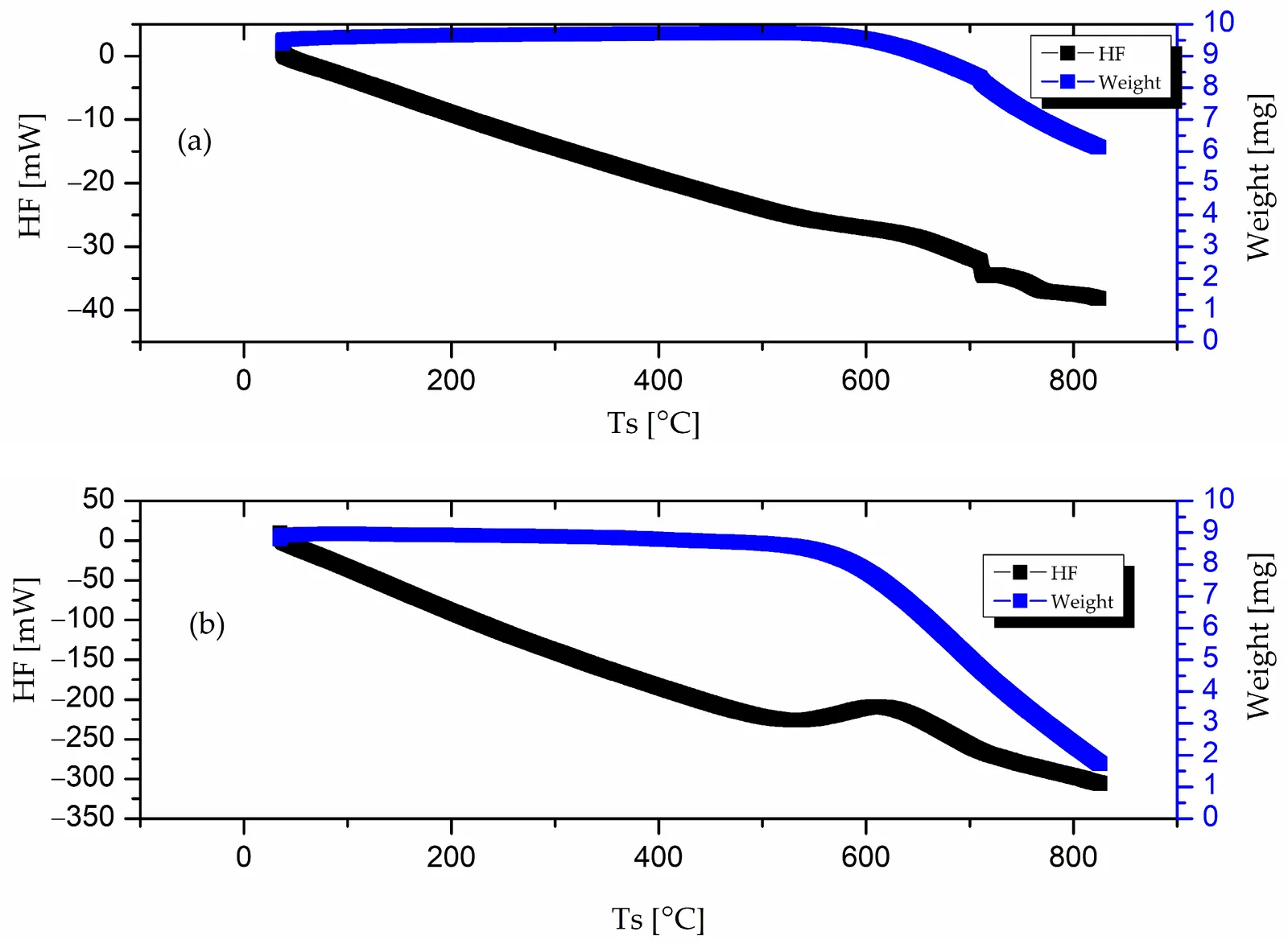
TG/DSC curves of (**a**) pristine MWCNT and (**b**) MWCNT-MAA.

Thermogravimetric and differential scanning calorimetry (TG–DSC) analyses were performed to further investigate the thermal behavior of pristine MWCNTs and MAA-treated MWCNTs. The corresponding TG–DSC curves are presented in Figure 4. Pristine MWCNTs exhibited relatively high thermal stability over a broad temperature range, with only a gradual decrease in mass at lower temperatures. A more pronounced mass loss was observed at temperatures above approximately 600 °C, which can be attributed to the thermal degradation of the carbon nanotube framework under the applied nitrogen atmosphere. Tables S1 and S2 present the selected temperature ranges, corresponding sample weights, and percentage mass losses. For MWCNTs, the first more pronounced mass loss of 12.78% occurred at 712 °C, followed by a mass loss of 26.16% at 771 °C and a final mass loss of 31.09% at 800 °C. For MWCNT-MAA, a mass loss of 18.31% was observed at 626 °C, whereas MWCNTs showed only a 0.56% mass loss at the same temperature. This was followed by a mass loss of 35.84% at 679 °C for MWCNT-MAA, while for MWCNTs, a comparable final mass loss (31.09%) was reached only at 800 °C. Furthermore, MWCNT-MAA showed a mass loss of 65.55% at 771 °C, followed by a final mass loss of 73.46% at 800 °C. The increased mass loss compared with pristine MWCNTs indicates the presence of an additional thermally labile organic fraction associated with the MWCNT surface. The DSC/heat-flow curves also revealed differences between the two materials. Pristine MWCNTs displayed a relatively gradual change in heat flow, followed by a more pronounced thermal event at higher temperatures, corresponding to the degradation of the carbonaceous material. After MAA treatment, the thermal profile was altered, with additional changes in the heat-flow signal occurring in the temperature region where decomposition of the introduced organic component is expected.

### 3.2. Optimization of the Membrane Composition for the MWCNT-MAA-MIP Sensor

The potentiometric response of the MWCNT-MAA-MIP sensor is related to the phase-boundary potential established at the membrane–solution interface. The response is associated with the partitioning and interaction of protonated TBZ with the recognition sites in the MIP-containing membrane. The measured potential depends on the activity of TBZ^+^ in the solution and follows the Nernst equation:

*E* = *E*^0^ + *S*·log *a*(TBZ^+^), (1)

where *E* is the measured electrode potential, *E*^0^ is the standard electrode potential, *S* is the slope obtained from the calibration curve, and *a*(TBZ^+^) is the activity of the protonated TBZ species. The theoretical Nernstian slope for a monovalent ion (TBZ^+^) at 25 °C is 59.2 mV/decade of activity.

The potentiometric characteristics of the prepared sensors were determined from calibration curves obtained by plotting the measured potentials against the logarithm of the calculated TBZ activity. The ionic strength was calculated using the following equation:

(2) $$I=0.5\;\sum c_{i}z_{i}^{2},$$

where *I* is the ionic strength, *c_i_* is the molar concentration of ion *i*, and *z_i_* is its charge. Considering the p*K*a value of TBZ and the measurement pH of 2.6, TBZ is predominantly present in its protonated form under these conditions [47]. Therefore, the analytical TBZ concentration was used as an approximation of the TBZ^+^ concentration. The small amount of HCl used for pH adjustment does not significantly affect the ionic strength and, consequently, has no significant effect on the calculated TBZ activities or the resulting calibration slopes.

The activity coefficients (*γ_i_*) were calculated using the Davies equation, and the activity was calculated as:

*a_i_* = *γ_i_c_i_*, (3)

where *a_i_* is the activity of ion *i*. Linear regression analysis was used to calculate the slope, linear range, and correlation coefficient. The LOD was determined from the intersection of the extrapolated linear portions of the potentiometric calibration curve according to the IUPAC recommendations [48], while the useful concentration range was defined from the linear response region of the calibration curve. The obtained results and statistical parameters are presented in the corresponding tables and figures.

#### 3.2.1. Influence of the Plasticizer Type on the Potentiometric Response

The plasticizer plays a key role in the PVC membrane of ion-selective sensors because it affects the physical properties of the membrane by increasing the flexibility of the polymer matrix and providing the formation of a homogeneous organic phase in which all membrane components are uniformly dispersed [49,50]. A suitable plasticizer contributes to the development of a stable membrane structure, while preserving the flexibility of the matrix and allowing access to the binding sites formed within the MIP structure. Since the type and amount of plasticizer can influence the response and analytical characteristics of the sensor membrane, four plasticizers (DS, *o*-NPOE, BEHS, and DBP) were investigated in this study, selected based on their suitability confirmed in a previous study [41].

**Figure 5:**
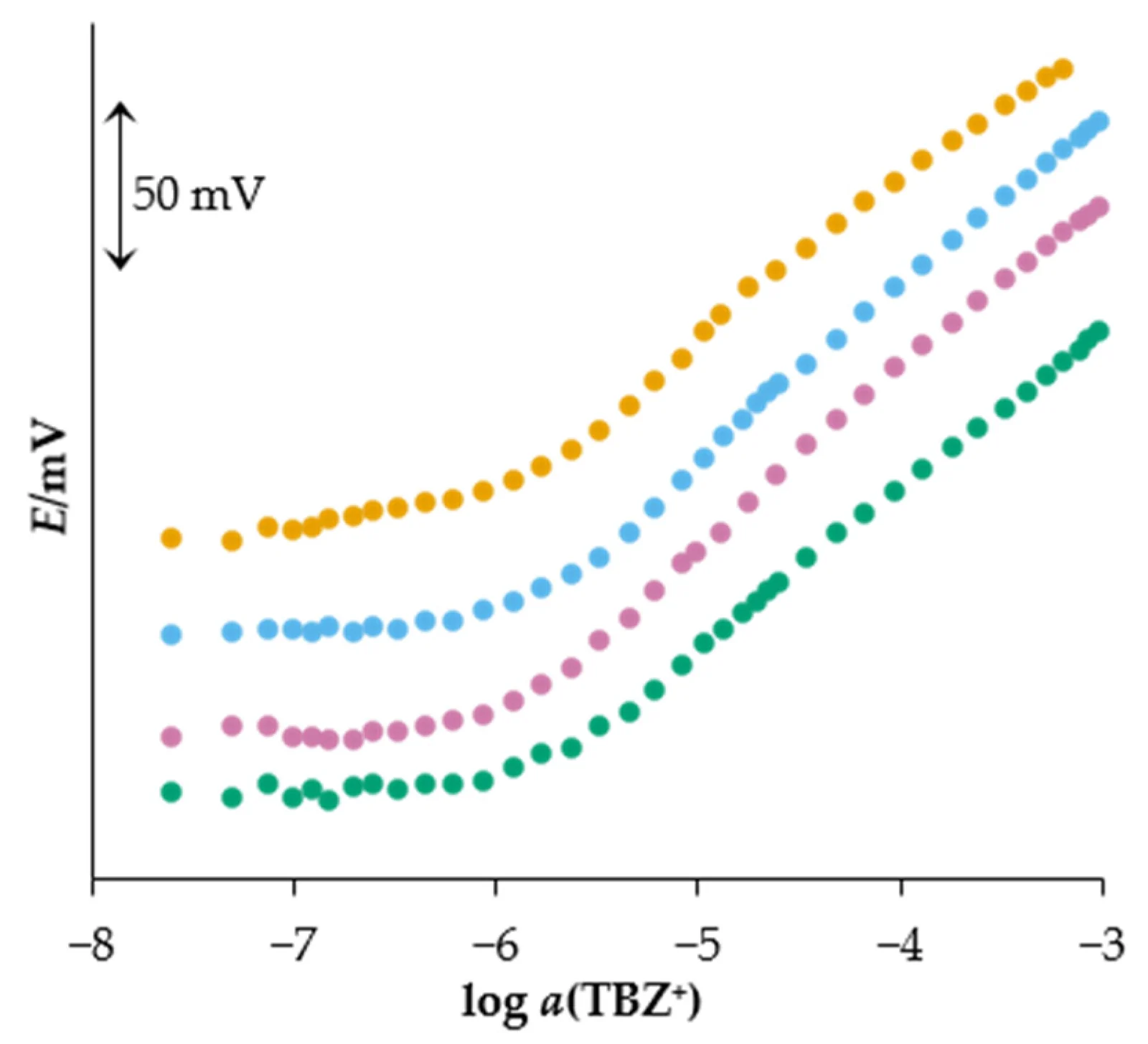
Responses to TBZ obtained using the MWCNT-MAA-MIP sensors with a different plasticizer (● BEHS, ● DS, ● DBP, and ● *o*-NPOE). Here and in further figures, some curves are displaced laterally and/or vertically for clarity.

In all prepared membranes, the MWCNT-MAA-MIP content was 3 wt%, while the MWCNT content was 1 wt%. The plasticizer-to-PVC ratio was 2:1 (63.83 and 32.12 wt%, respectively). The potentiometric responses of the sensors prepared with different plasticizers are presented in Table 1 and graphically in Figure 5.

The sensor responses toward TBZ were investigated in the concentration range from 2.5·10^−8^ to 1.0·10^−3^ M. The results showed that the sensor prepared with DBP as the plasticizer exhibited a Nernstian response, with a slope of 57.3 ± 0.8 mV/decade of activity. In addition, this sensor showed a wide linear range from 8.6·10^−7^ to 1.0·10^−3^ M.

The sensors prepared with DS, *o*-NPOE, and BEHS showed lower slope values of 55.2, 51.6, and 48.1 mV/decade of activity, respectively, indicating a sub-Nernstian response. Based on its Nernstian response and wide linear range, DBP was selected for further investigations.

#### 3.2.2. Influence of the MWCNT-MAA-MIP Content on the Potentiometric Response

The amount of MWCNT-MAA-MIP in the membrane composition can affect the potentiometric response by influencing the availability of recognition sites within the MIP structure and the interaction with the analyte. Therefore, different amounts of MWCNT-MAA-MIP were investigated to determine their effect on the sensor response and to identify the optimal content for membrane preparation.

**Figure 6:**
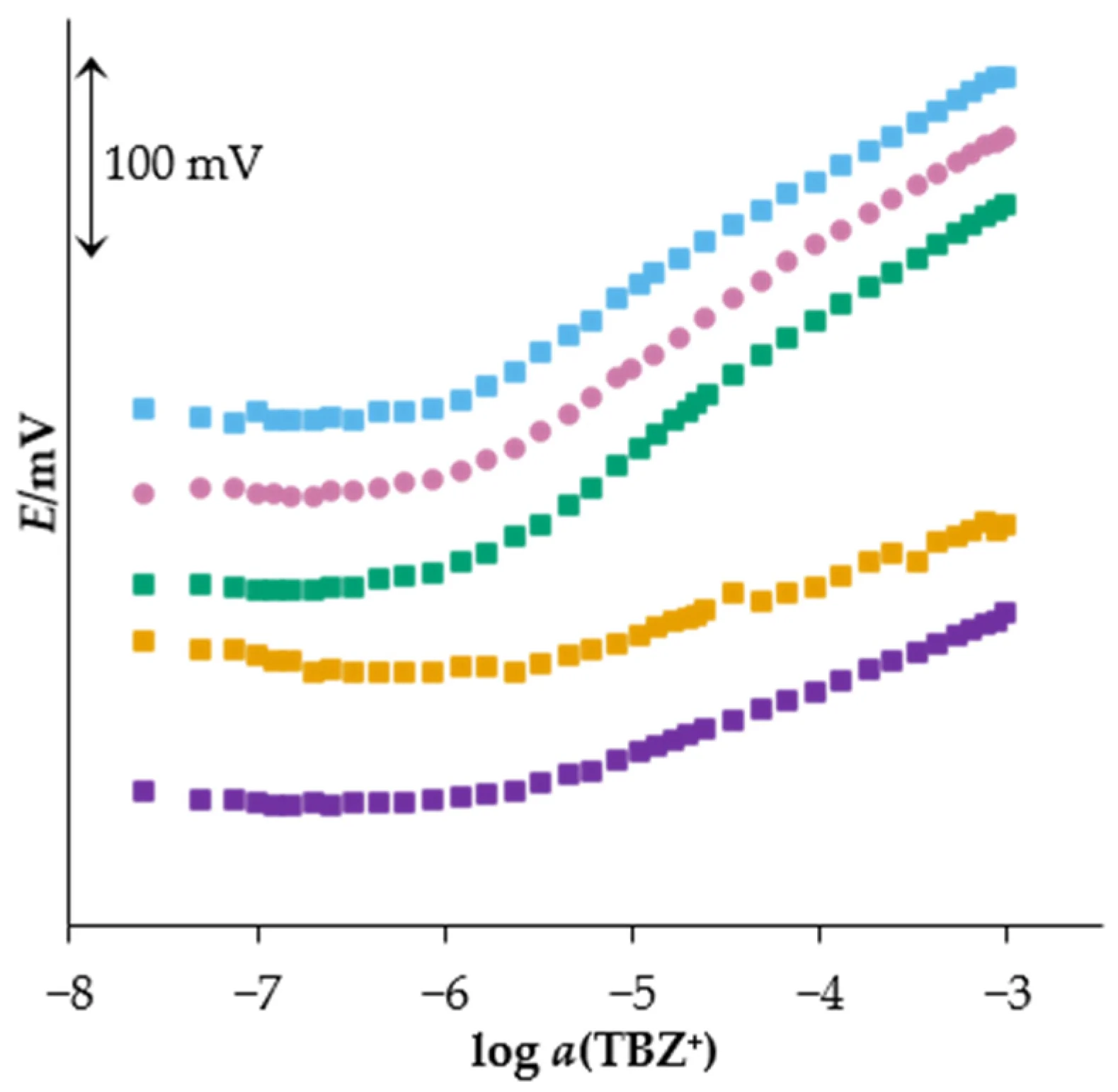
Responses to TBZ obtained using the MWCNT-MAA-MIP sensors with a different content of sensor material (■ 1, ● 3, ■ 5, ■ 7, and ■ 10 wt%).

The influence of different amounts of MWCNT-MAA-MIP on the potentiometric response of the TBZ sensor was investigated using membranes containing 1, 3, 5, 7, and 10 wt% of MWCNT-MAA-MIP. In all investigated sensors, the DBP-to-PVC ratio was 2:1, while the content of MWCNTs was 1 wt%. The results are presented in Table 1 and Figure 6.

Among the investigated membranes, the sensor containing 3 wt% of MWCNT-MAA-MIP exhibited the widest linear range and a slope closest to the theoretical Nernstian value (57.3 ± 0.8 mV/decade of activity). Although the sensor containing 5 wt% of MWCNT-MAA-MIP showed a high slope value (61.9 ± 1.7 mV/decade of activity), which was also close to the theoretical Nernstian slope, its linear range was narrower. The sensors containing other amounts of MWCNT-MAA-MIP showed lower slope values and narrower linear ranges. This can be related to changes in the number of available recognition sites within the MIP structure, which may influence the interaction between the analyte and the membrane and result in a lower potentiometric response.

Based on these results, the sensor containing 3 wt% of MWCNT-MAA-MIP with DBP as the plasticizer was selected for further investigations.

#### 3.2.3. Influence of the MWCNTs Content on the Potentiometric Response

Since the incorporation of MWCNTs can affect the properties and composition of the sensing membrane, different amounts of MWCNTs were investigated to evaluate their influence on the potentiometric response and to select the appropriate membrane composition.

**Figure 7:**
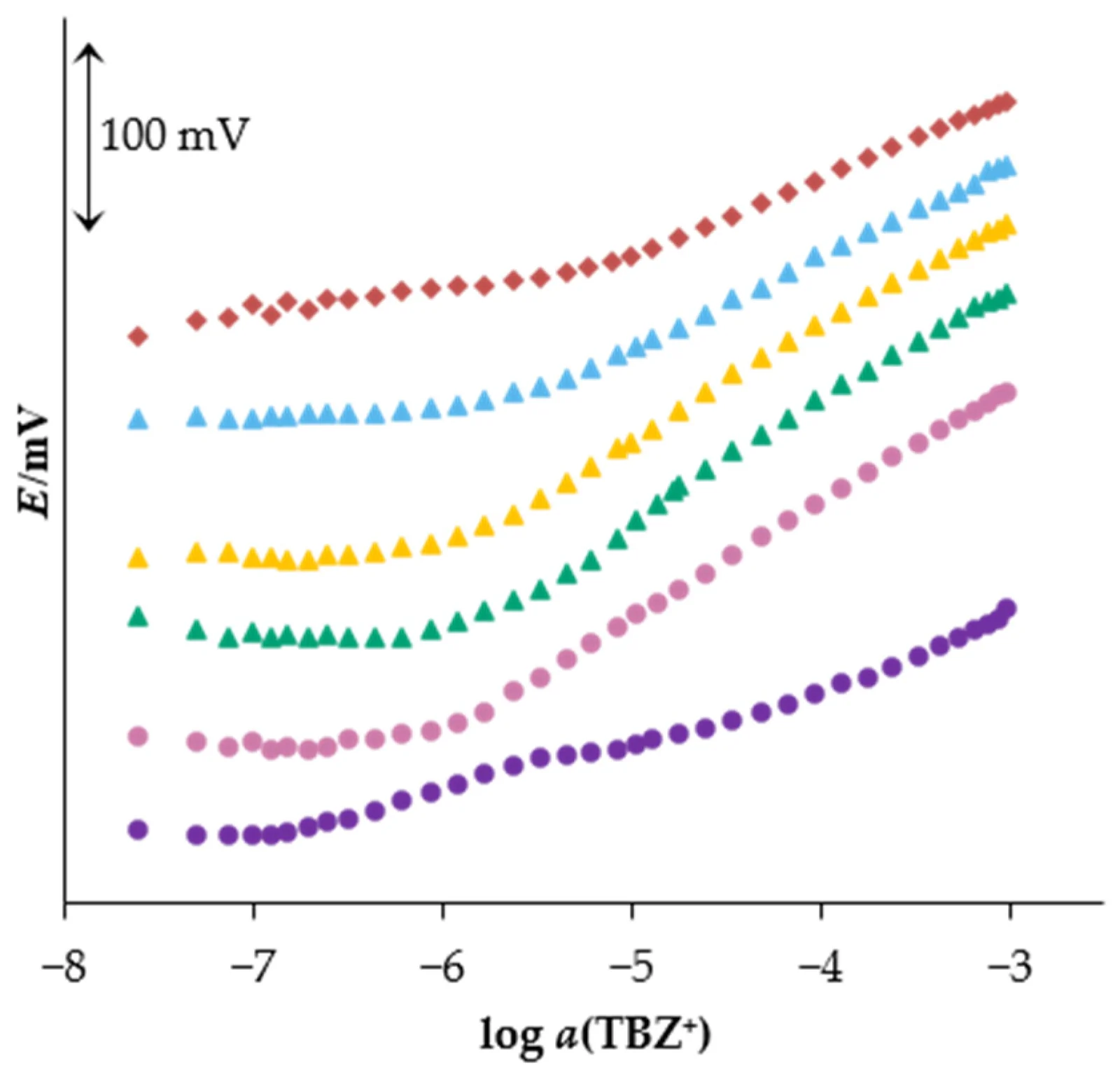
Responses to TBZ obtained using the MWCNT-MAA-MIP sensors with a different content of MWCNTs (◆ 0, ▲ 0.5, ▲ 1, ● 2, and ▲ 3 wt%) and using a NIP-based sensor (●).

The influence of different amounts of MWCNTs in the sensor membrane on the potentiometric response of the MWCNT-MAA-MIP sensor was investigated using sensors containing 0, 0.5, 1, 2, and 3 wt% of MWCNTs in the membrane. In all prepared sensors, the DBP-to-PVC ratio was 2:1, while the content of MWCNT-MAA-MIP was 3 wt%. The membrane containing 0 wt% of additional MWCNTs, prepared with the same MWCNT-MAA-MIP content and under the same experimental conditions, served as a control for evaluating the contribution of additionally incorporated MWCNTs to the potentiometric response. The results are presented in Table 1 and Figure 7.

The sensor containing 2 wt% of MWCNTs exhibited the Nernstian slope (59.7 ± 0.5 mV/decade of activity), together with the lowest LOD, the widest linear range, and the highest linearity of the calibration curve (R^2^ = 0.9997). Although the sensor containing 3 wt% of MWCNTs showed a slightly higher slope value (62.0 ± 1.8 mV/decade of activity), it exhibited a lower R^2^ value (0.9961), indicating a less favorable calibration response. The control sensor without additionally incorporated MWCNTs exhibited a considerably lower slope of 39.0 ± 1.1 mV/decade of activity and a higher LOD of 1.7·10^−6^ M. Sensors containing lower MWCNT content showed sub-Nernstian slope values. The improved potentiometric characteristics observed upon the incorporation of additional MWCNTs demonstrate their contribution to the sensor response. Based on these results, the sensor containing DBP as the plasticizer, 3 wt% of MWCNT-MAA-MIP, and 2 wt% of MWCNTs was selected for further investigations. The obtained results show that the amount of MWCNTs incorporated into the membrane affects the potentiometric characteristics of the sensor. At the highest investigated MWCNT content (3 wt%), reduced membrane homogeneity was visually observed during membrane preparation, which may have contributed to the less favorable overall analytical characteristics. Among the investigated MWCNT contents, 2 wt% provided the best overall combination of analytical parameters. After selection of the optimal membrane composition, two additional sensors were independently prepared using the same optimized composition to evaluate sensor-to-sensor reproducibility. The three independently prepared sensors (S1–S3) showed comparable potentiometric characteristics, with a mean slope of 59.7 mV/decade of activity, an SD of 0.15 mV/decade of activity, and an RSD of 0.26% across the three sensors. All three sensors showed the same LOD (3.2·10^−7^ M) and useful concentration range (6.2·10^−7^–1.0·10^−3^ M), as shown in Table 1. The results presented in the figures throughout the manuscript were obtained using sensor S1. For each of the independently prepared sensors S1–S3, five replicate measurements were performed using the same sensor to evaluate measurement repeatability. The results obtained with S1–S3 were compared to evaluate sensor-to-sensor reproducibility.

To examine the contribution of MIP to the sensor response, a sensor containing 3 wt% MWCNT-MAA-NIP was prepared. The remaining membrane components were identical to those used in the selected MWCNT-MAA-MIP sensor. The response of the MWCNT-MAA-NIP sensor is presented in Figure 7, showing a lower potentiometric response than the corresponding MIP-based sensor, which supports the role of molecular imprinting in the recognition of TBZ. As shown in Table 1, the NIP-based sensor exhibited a lower slope, poorer linearity, and a considerably narrower useful concentration range than the corresponding MIP-based sensor.

### 3.3. Dynamic Response Time of the MWCNT-MAA-MIP Sensor

The dynamic response time was defined as the time required for the sensor potential to reach 95% of the final stable value after each change in analyte concentration.

**Figure 8:**
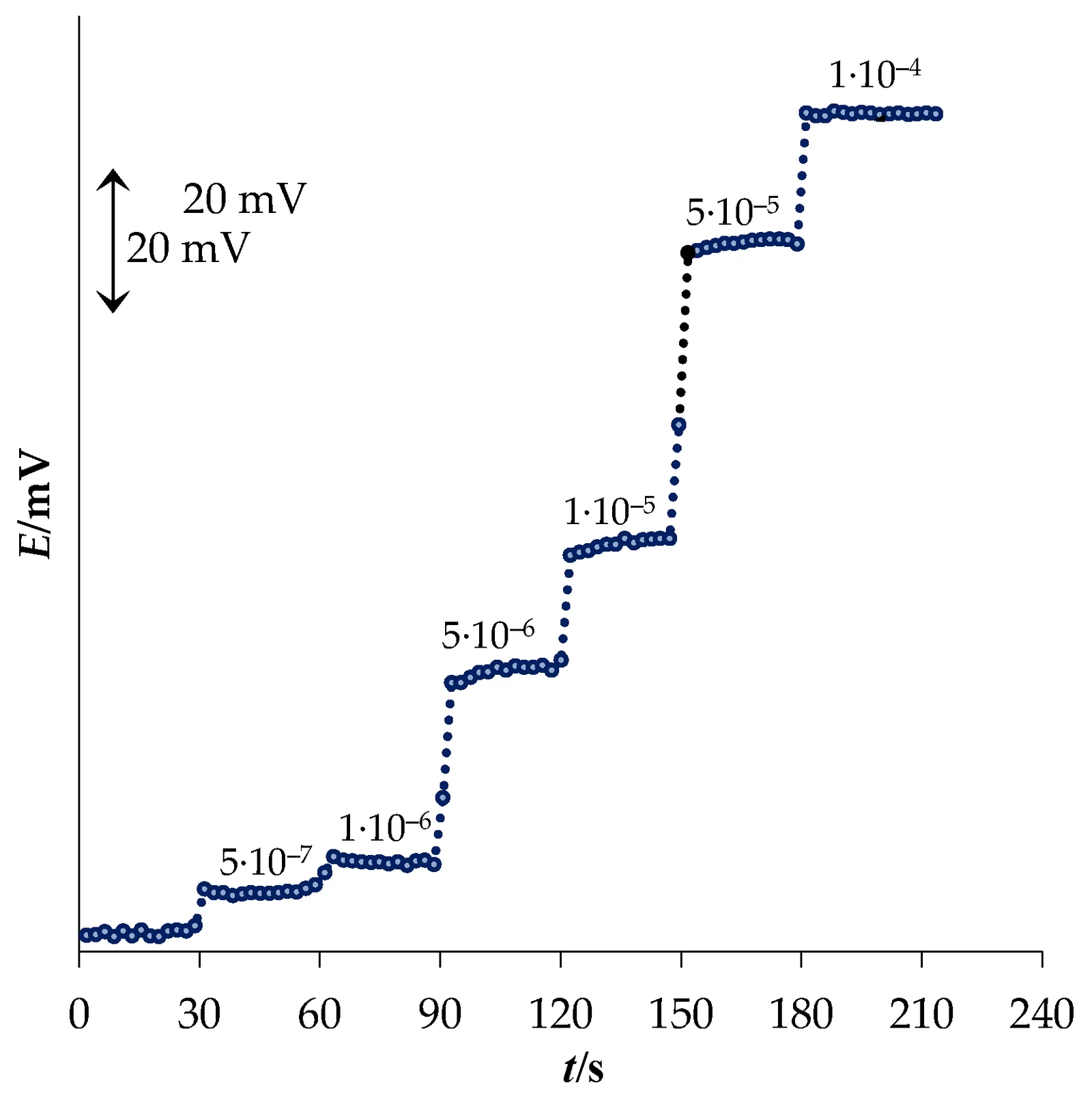
Dynamic response time of the MWCNT-MAA-MIP sensor.

As can be seen in Figure 8, the MWCNT-MAA-MIP sensor showed a very fast dynamic response time with an average response time of 4 s in the range from 0 to 1·10^−4^ M, with a time interval of 30 s.

### 3.4. Signal Drift and Lifetime of the MWCNT-MAA-MIP Sensor

Sensor drift refers to the gradual change in sensor potential over time under constant measurement conditions.

The signal drift of the MWCNT-MAA-MIP sensor was determined in a TBZ solution with a concentration of 1·10^−3^ M at room temperature. The drift value was calculated using linear regression analysis and described by Equation (4), with a drift rate of −0.72 mV/h.

*E* = −0.00020 ∙ *t*(s) + 80.43 (4)

**Figure 9:**
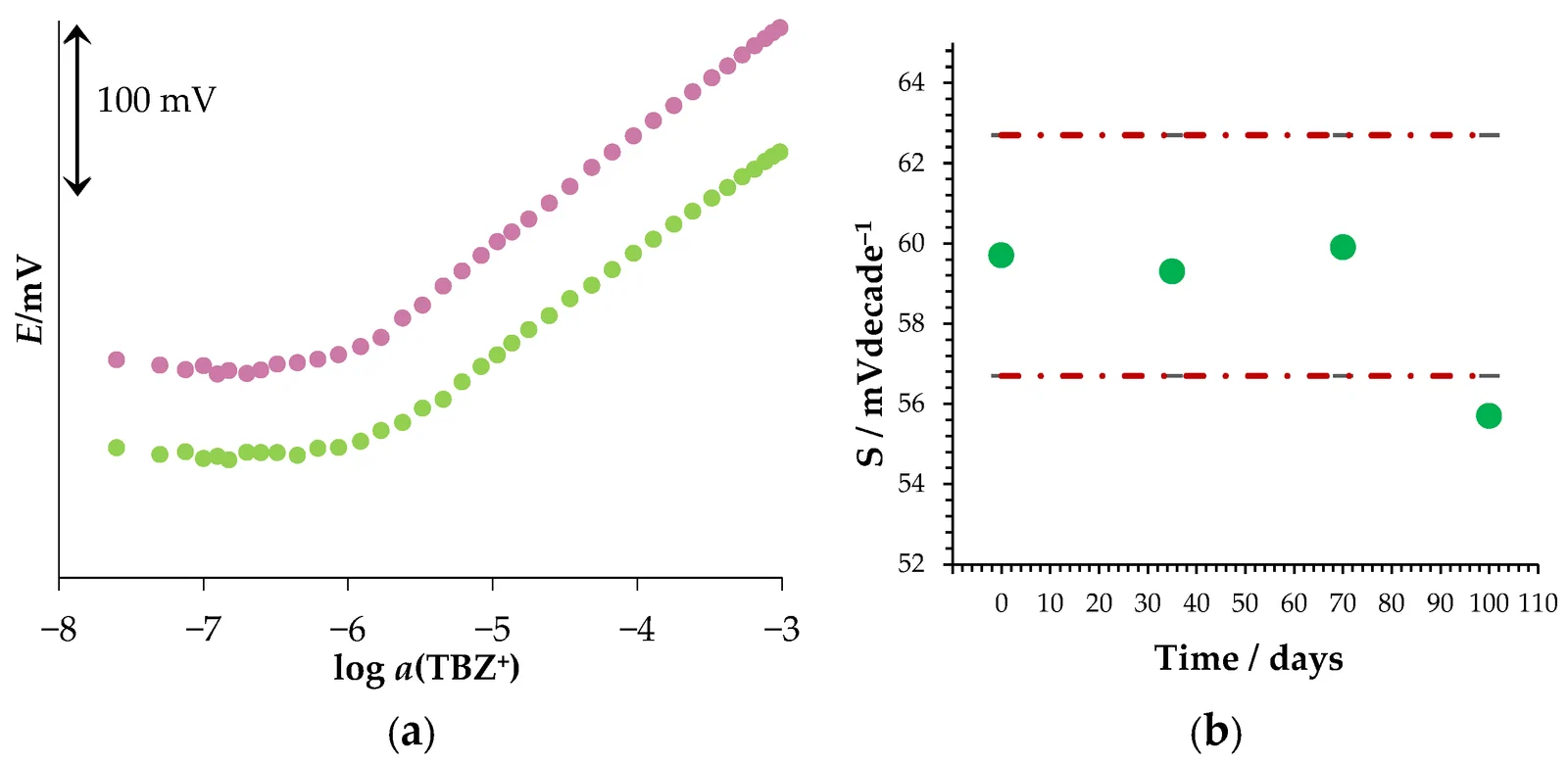
Long-term response stability of the MWCNT-MAA-MIP sensor: (**a**) potentiometric responses toward TBZ obtained during sensor characterization and after approximately 100 days (● sensor during characterization, ● sensor after approximately 100 days of daily measurements); (**b**) calibration slope as a function of use time (● represent the initial sensor slope and slopes measured after 35, 70, and 100 days, while red dashed lines indicate the ±5% acceptance limits).

The lifetime of the MWCNT-MAA-MIP sensor was evaluated by monitoring the calibration slope during daily measurements. The sensor was considered stable as long as the calibration slope remained within ± 5% of the value determined during sensor characterization. The end of the sensor lifetime was defined as the point at which the calibration slope deviated by more than 5% compared with the value determined during sensor characterization. As shown in Figure 9b, representative slope values remained within the ± 5% acceptance limits after 35 and 70 days, whereas after approximately 100 days the slope decreased to 55.7 mV/decade of activity, falling below the lower acceptance limit. The corresponding potentiometric responses obtained during sensor characterization and after approximately 100 days are shown in Figure 9a.

### 3.5. The Influence of pH

**Figure 10:**
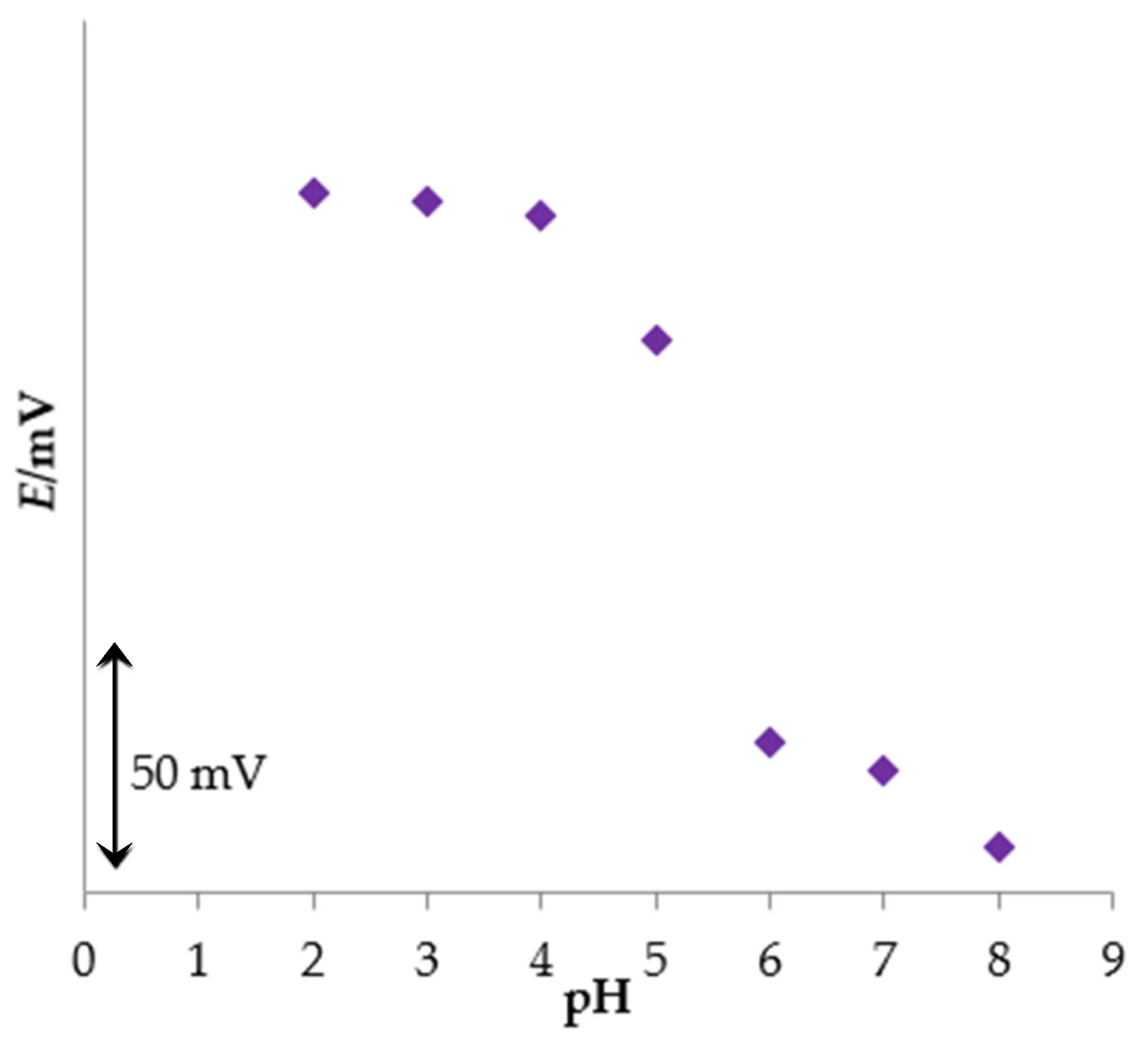
The influence of the pH value on the potentiometric response of the MWCNT-MAA-MIP sensor.

The influence of pH on the potentiometric response of the MWCNT-MAA-MIP sensor toward TBZ (*c* = 1·10^−3^ M) was investigated in the pH range from 2 to 8. The results are presented in Figure 10.

The MWCNT-MAA-MIP sensor showed stable potential in the pH range from 2 to 4.

At higher pH values, a decrease in potential was observed, which can be attributed to the deprotonation of TBZ in the aqueous phase at the membrane/solution interface, resulting in reduced interaction with the sensor material in the membrane.

The relatively narrow pH range of stable response does not limit the application of the sensor, since TBZ is predominantly present in its protonated form under acidic conditions (approximately pH 3 and below), which is favorable for its potentiometric determination.

### 3.6. The Selectivity of the MWCNT-MAA-MIP Sensor

Selectivity is an important characteristic of ion-selective membranes. Membranes are generally not selective only toward the target analyte, since other species can also affect the measured potential. Therefore, selectivity needs to be determined to assess whether measurements can be performed without a significant influence of interfering species on the potential of the MWCNT-MAA-MIP sensor and to evaluate the possibility of using the sensor for TBZ determination in more complex analytical matrices, such as real samples.

The selectivity coefficients were calculated using the Nikolsky–Eisenman equation:

(5) $$E=E^{{}^{\circ}}+\frac{2.303\mathrm{RT}}{z_{A}F}\log\left[a_{A}+\sum_{B=1}^{N}K_{A,B}^{\mathrm{pot}}\;a_{B}^{\frac{z_{A}}{z_{B}}}\;\right],$$

where *a*_A_ is the activity of the analyte ion, *z*_A_ is its charge, $K_{A,B}^{\mathrm{pot}}$ is the potentiometric selectivity coefficient of the MWCNT-MAA-MIP sensor for ion B relative to ion A, *a*_B_ is the activity of the interfering ion B, and *z*_B_ is its charge. The lower the selectivity coefficient, the better the selectivity of the electrode.

The $K_{A,B}^{\mathrm{pot}}$ values for the MWCNT-MAA-MIP sensor were determined using the fixed interference method, where the potentiometric response of the MWCNT-MAA-MIP sensor was measured in solutions containing a fixed concentration of interfering ion (*c* = 1·10^−2^ M, except for imazalil, and fuberidazole, for which *c* = 2.5·10^−4^ M was used, and carbendazim, for which *c* = 5·10^−5^ M was used due to its limited solubility).

The calculated $K_{A,B}^{\mathrm{pot}}\;$ values for the potentially interfering species expected in real samples are presented in Table 2. Based on the obtained results, the MWCNT-MAA-MIP sensor exhibited good selectivity toward TBZ, indicating its potential for reliable TBZ determination in real samples.

### 3.7. Comparison with Previously Developed TBZ Potentiometric Sensors

The analytical performance, selectivity, and applicability of the MWCNT-MAA-MIP sensor were compared with those of previously reported potentiometric sensors for TBZ determination (Table S3). These sensors represent different sensing approaches for TBZ determination.

As shown in Table S3, the slope, LOD, and useful concentration range of the present sensor are comparable to those of the previously reported TBZ potentiometric sensors. Compared with previously reported sensors, the present sensor showed the best agreement with the theoretical Nernstian slope, the shortest response time, and the lowest potential drift, while maintaining a lifetime of approximately 100 days. The present sensor also showed good selectivity toward the investigated interfering species and was applied to TBZ determination in prepared orange, lemon, and banana peel extracts. The obtained analytical recoveries (104.2–107.6%) were comparable to those reported for the previously developed TBZ potentiometric sensors.

### 3.8. Applicability of the MWCNT-MAA-MIP Sensor

To evaluate the accuracy and precision of the newly developed MWCNT-MAA-MIP sensor for TBZ determination, standard TBZ solutions were analyzed using direct potentiometry and the Gran method. Direct potentiometric measurements were performed in TBZ solutions of known concentrations, ranging from 1·10^−5^ M to 1·10^−3^ M, and the analyte concentration was determined based on the calibration curve obtained by plotting the potential against the logarithm of activity. The Gran method, originally described by Gran [51], was applied as an extension of the standard addition method. For this purpose, ten incremental additions of the standard TBZ solution (*c* = 2·10^−3^ M) were added to each analyzed standard solution, and the corresponding potentials were measured.

The results obtained (Table 3) were compared to evaluate the accuracy of TBZ determination. Considering the low concentration of the analyzed solutions, both methods showed acceptable results. Slightly better results were obtained using the Gran method, which was expected since multiple standard additions allow more accurate determination of the analyte concentration. Therefore, the Gran method was selected for TBZ determination in prepared fruit-peel extracts, applying the standard addition principle to evaluate the matrix influence on TBZ determination.

TBZ concentration was determined using the Gran method according to the following equation:

(6) $$10^{\frac{E_{1}-E_{0}}{S}}\left(V_{0}+V_{s}\right)=V_{0}+\frac{1}{c_{x}}c_{s}V_{s},$$

where *E*_0_ and *E*_1_ are the potentials measured before and after standard addition, respectively, *V*_0_ is the initial sample volume, *V*_s_ is the cumulative volume of incrementally added standard solution, *c*_s_ is the concentration of the standard solution, and *c*_x_ is the initial TBZ concentration in the sample. The term (*V*_0_ + V_s_) accounts for the change in total solution volume during the successive standard additions.

Since TBZ is commonly used as a post-harvest treatment for oranges, lemons, and bananas, these fruits were selected as real samples for TBZ determination. The three independently prepared sensors (S1–S3) showed comparable potentiometric characteristics; therefore, all measurements of the prepared fruit-peel extracts were performed using sensor S1. The initial TBZ concentration in the prepared fruit-peel extracts was determined using the Gran method, with ten incremental additions of TBZ standard solution to 15 mL of each extract. For the fortified extracts, measurements were performed by adding 10 mL of TBZ standard solution to 15 mL of the extract, followed by ten incremental additions of the same standard solution. The initial addition of 10 mL of standard solution reduced the extract fraction to 60% of the total volume before the incremental additions. The total volume of the ten incremental additions was 7.5 mL, and the corresponding volume changes were accounted for in the Gran calculation.

To express the sensor LOD on a peel-mass basis, the experimentally determined LOD (3.2·10^−7^ M) was converted using the initial peel mass (100 g) and the respective extraction volumes. The corresponding values were 0.128 mg kg^−1^ for orange and lemon peel and 0.115 mg kg^−1^ for banana peel. A separate LOQ was not experimentally determined during sensor characterization. The lower limit of the useful concentration range (6.2·10^−7^ M) corresponded to 0.248 mg kg^−1^ for orange peel, 0.247 mg kg^−1^ for lemon peel, and 0.223 mg kg^−1^ for banana peel. The obtained results (Table 4) showed recovery values of 107.6% for orange, 104.2% for banana, and 106.0% for lemon, with the corresponding SD values reported separately for each sample. Since TBZ was added after extraction, the obtained analytical recoveries reflect the accuracy of the potentiometric determination in the fruit-peel extracts, rather than the recovery of TBZ during the extraction step.

## 4. Conclusions

In this study, a new potentiometric sensor based on an MWCNT-modified MIP was developed and optimized. The prepared materials were characterized by FT-IR spectroscopy and TG–DSC analysis. The membrane composition was optimized using four different plasticizers: DBP, *o*-NPOE, BEHS, and DS. Membranes containing different amounts of MWCNT-MAA-MIP (1, 3, 5, 7, and 10 wt%) and MWCNTs (0, 0.5, 1, 2, and 3 wt%) were prepared and tested. The best performance was obtained for the membrane containing DBP as a plasticizer, 3 wt% MWCNT-MAA-MIP, and 2 wt% MWCNTs. The developed sensor showed a Nernstian slope of 59.7 mV/decade of activity, a detection limit of 3.2·10^−7^ M, and a wide useful concentration range (6.2·10^−7^–1·10^−3^ M). The response time was 4 s, and the sensor showed good selectivity toward TBZ. The accuracy of TBZ determination was confirmed using solutions with known TBZ concentrations, while the standard addition method was applied to evaluate TBZ determination in prepared fruit-peel extracts. The sensor was applied to TBZ determination in prepared orange, banana, and lemon peel extracts. Compared with the previously developed TBZ potentiometric sensors, the present sensor showed the best agreement with the theoretical Nernstian slope, together with a shorter response time and improved potential stability, while retaining comparable LOD and useful concentration range and a lifetime of approximately 100 days.

## Figures and Tables

**Table 1:** Response characteristics of the MWCNT-MAA-MIP sensors with different plasticizers, different contents of the sensor material, and different contents of MWCNTs to TBZ ^1^.

Plasticizer	Content of the Sensor Material (%)	Content of the MWCNT (%)	Slope (mV/Decade of Activity)	R^2^	LOD (M)	Useful Conc. Range (M)
DS	3	1	55.2 ± 0.5	0.9997	8.6·10^−7^	1.2·10^−6^–1.0·10^−3^
*o*-NPOE	3	1	51.6 ± 0.5	0.9996	1.2·10^−6^	2.4·10^−6^–1.0·10^−3^
BEHS	3	1	48.1 ± 1.0	0.9988	1.2·10^−6^	2.4·10^−6^–1.0·10^−3^
DBP	3	1	57.3 ± 0.8	0.9994	6.2·10^−7^	8.6·10^−7^–1.0·10^−3^
DBP	1	1	31.7 ± 3.9	0.9813	1.7·10^−6^	2.4·10^−6^–2.5·10^−5^
DBP	5	1	61.9 ± 1.7	0.9964	1.2·10^−6^	1.7·10^−6^–1.0·10^−3^
DBP	7	1	53.1 ± 1.0	0.9979	8.6·10^−7^	1.2·10^−6^–1.0·10^−3^
DBP	10	1	33.6 ± 0.6	0.9986	1.7·10^−6^	2.4·10^−6^–1.0·10^−3^
DBP	3	0	39.0 ± 1.1	0.9962	1.7·10^−6^	2.4·10^−6^–1.0·10^−3^
DBP	3	0.5	48.0 ± 0.5	0.9995	8.6·10^−7^	1.7·10^−6^–1.0·10^−3^
DBP	3	3	62.0 ± 1.8	0.9961	8.6·10^−7^	1.2·10^−6^–1.0·10^−3^
DBP (S1)	3	2	59.7 ± 0.5	0.9997	3.2·10^−7^	6.2·10^−7^–1.0·10^−3^
DBP (S2)	3	2	59.6 ± 0.4	0.9990	3.2·10^−7^	6.2·10^−7^–1.0·10^−3^
DBP (S3)	3	2	59.9 ± 0.5	0.9995	3.2·10^−7^	6.2·10^−7^–1.0·10^−3^
DBP	3 (NIP)	2	29.7 ± 2.4	0.9718	3.2·10^−7^	6.2·10^−7^–4.5·10^−6^

^1^ Average of 5 determinations ± confidence limits (*p* = 0.95). S1, S2, and S3 denote three independently prepared sensors with the same optimized membrane composition.

**Table 2:** Potentiometric selectivity coefficients of the MWCNT-MAA-MIP sensor obtained by the fixed interference method.

Potential Interferents	$K_{A,B}^{\mathrm{pot}}$
Ammonium	1.27·10^−4^
Sodium	1.02·10^−3^
Calcium	3.83·10^−6^
Magnesium	2.50·10^−4^
Potassium	9.32·10^−4^
Copper	3.79·10^−3^
Zinc	7.71·10^−5^
Lithium	7.94·10^−5^
Iron(III)	9.80·10^−4^
Iron(II)	2.56·10^−4^
Imazalil	3.98·10^−5^
Fuberidazole	9.38·10^−5^
Carbendazim	9.33·10^−5^

**Table 3:** Comparative results of TBZ determination in standard solutions using direct potentiometry and the Gran method with the MWCNT-MAA-MIP sensor ^1^.

TBZ Added [M]	TBZ Found [M] ± SD		Recovery [%]	
	Direct Potentiometry	Gran Method	Direct Potentiometry	Gran Method
1.00·10^−3^	1.03·10^−3^ ± 1.12·10^−5^	1.01·10^−3^ ± 2.68·10^−5^	102.9	100.7
5.00·10^−4^	4.89·10^−4^ ± 1.54·10^−6^	4.96·10^−4^ ± 1.70·10^−5^	97.9	99.2
1.00·10^−4^	1.04·10^−4^ ± 5.20·10^−7^	1.01·10^−4^ ± 1.06·10^−6^	103.6	101.4
5.00·10^−5^	4.79·10^−5^ ± 5.75·10^−7^	4.98·10^−5^ ± 1.48·10^−6^	95.8	99.7
1.00·10^−5^	9.42·10^−6^ ± 4.11·10^−8^	1.01·10^−5^ ± 7.76·10^−7^	94.2	100.8

^1^ Average of 3 determinations performed using sensor S1.

**Table 4:** Results of TBZ determination in prepared fruit-peel extracts of oranges, bananas, and lemons using the Gran method with the MWCNT-MAA-MIP sensor ^1^.

Sample	TBZ Found [M]	TBZ Found [mg/g of the Peel]	TBZ Added [mol]	TBZ Found [mol] ± SD	Analytical Recovery [%]
Oranges	6.54·10^−4^	0.261	2.00·10^−5^	2.14·10^−5^ ± 1.86·10^−7^	107.6
Bananas	2.66·10^−4^	0.096	2.00·10^−5^	2.08·10^−5^ ± 6.62·10^−7^	104.2
Lemons	5.38·10^−4^	0.214	2.00·10^−5^	2.12·10^−5^ ± 3.05·10^−7^	106.0

^1^ Average of 3 determinations performed using sensor S1.

## Data Availability

The data presented in this study are available upon request.

## Supplementary Materials

The following supporting information can be downloaded at: https://www.mdpi.com/article/10.3390/s26206474/s1, Table S1: Individual mass-loss stages at corresponding temperature intervals for MWCNT, Table S2: Individual mass-loss stages at corresponding temperature intervals for MWCNT-MAA, Table S3. Comparison of the analytical characteristics of the developed sensor with previously reported TBZ potentiometric sensors.

## Abbreviations

The following abbreviations are used in this manuscript:

TBZ	Thiabendazole
MIP	Molecularly imprinted polymer
THF	Tetrahydrofuran
PVC	Polyvinyl chloride
*o*-NPOE	*o*-nitrophenyloctylether
BEHS	Bis(2-ethylhexyl) sebacate
DBP	Dibutyl phthalate
DS	Dibutyl sebacate
MAA	Methacrylic acid
TRIM	Trimethylolpropane triacrylate
