# Supplementary material for: A Potentiometric Sensor Based on a Molecularly Imprinted Polymer Modified with Multi-Walled Carbon Nanotubes for the Selective Determination of Thiabendazole Residues in Fruit Peels
Source: Sensors (Basel). 2026 Oct 13;26(20):6474. doi: 10.3390/s26206474 (PMC13709216; doi:10.3390/s26206474)
Supplement: Supplementary file 1 [file sensors-26-06474-s001.zip › sensors-4587990-supplementary.pdf]

Article

A Potentiometric Sensor Based on a Molecularly Imprinted Polymer Modified with Multi-Walled Carbon Nanotubes for the Selective Determination of Thiabendazole Residues in Fruit Peels

Mirela Samardžić, Andrea Dandić, Izabella Doris Panić and Mateja Budetić *

Department of Chemistry, Josip Juraj Strossmayer University of Osijek, Cara Hadrijana 8/A, 31000 Osijek, Croatia; mirelas@kemija.unios.hr (M.S.); andreajuric@kemija.unios.hr (A.D.); doris.panic123@gmail.com (I.D.P.)

* Correspondence: mbudetic@kemija.unios.hr

Table S1. Individual mass-loss stages at corresponding temperature intervals for MWCNT.

Ts [◦C]	Weight [mg]	% of mass loss
36.9481	9.38911	0
626.848	9.33662	0.56
630.527	9.3032	0.91
640.218	9.2085	0.92
660.263	8.98497	4.30
679.732	8.73773	6.93
712.124	8.18857	12.78
736.08	7.60618	18.98
771.141	6.93334	26.16
779.659	6.7888	27.69
786.06	6.68445	28.80
794.948	6.54575	30.28
798.889	6.4866	30.91
800.047	6.46967	31.09

Table S2. Individual mass-loss stages at corresponding temperature intervals for MWCNT-MAA.

Ts [◦C]	Weight [mg]	% of mass loss
34.6518	8.80008	0
626.303	7.18825	18.31
630.31	7.08391	19.50
640.147	6.81797	22.52
658.538	6.28947	28.53
679.708	5.6465	35.84
712.345	4.64231	47.25
736.037	3.97008	54.88
771.852	3.03176	65.55
779.398	2.8417	67.71
786.269	2.67173	69.64
794.989	2.45712	72.08
798.832	2.36359	73.14
800.007	2.33524	73.46

Table S3. Comparison of the analytical characteristics of the developed sensor with previously reported TBZ potentiometric sensors.

Sensor design	Slope (mV/decade of activity)	Useful range (M)	LOD (M)	Response time (s)	∣Drift∣ (mV/h)	Lifetime (days)	Selectivity	Sample	Analytical recovery
Liquid inner electrolyte ISE; TBZ^+–5-sulfosalicylate ion pair [25]	62.2 ± 0.9	$8.6 \cdot 10^{-7} – 1.0 \cdot 10^{-3}$	$3.2 \cdot 10^{-7}$	8 ($c = 5.0 \cdot 10^{-7} – 1.0 \cdot 10^{-4}$)	3.5	≈ 30	15 interferents (9 inorganic ions + 6 organic compounds)	peels of oranges, lemons, and bananas	102.4 – 107.0
Solid-state ISE; hybrid sensing material based on functionalized MWCNTs and TBZ^+ ($MWCNT\text{-}OSO_3^- TBZ^+$) [23]	60.4 ± 0.8	$8.6 \cdot 10^{-7} – 1.0 \cdot 10^{-3}$	$6.2 \cdot 10^{-7}$	8 ($c = 5.0 \cdot 10^{-7} – 1.0 \cdot 10^{-4}$)	1.7	≈ 90	16 interferents (10 inorganic ions + 6 organic compounds)	peels of oranges, lemons, bananas, clementines, and limes	95.1–110.0
Liquid inner electrolyte ISE; MIP-TBZ without MWCNTs [41]	60.8 ± 1.0	$6.2 \cdot 10^{-7} – 1.0 \cdot 10^{-3}$	$3.2 \cdot 10^{-7}$	6 ($c = 5.0 \cdot 10^{-7} – 1.0 \cdot 10^{-4}$)	1.4	≈ 100	11 interferents (9 inorganic ions + 2 organic compounds)	peels of oranges, lemons, and bananas	93.7 –109.2
Liquid inner electrolyte ISE; ion pair of TBZ with 12-molybdophosphoric acid [24]	30	$1.0 \cdot 10^{-5} – 1.0 \cdot 10^{-2}$	$1.00 \cdot 10^{-5}$	40 – 50 ($c = 10^{-3} – 10^{-2}$); 120 – 180 ($c = 10^{-6} – 10^{-4}$ M)	/	65	/	/	/
Liquid inner electrolyte ISE; MAA-treated MWCNTs incorporated during MIP synthesis + additional MWCNTs incorporated into the membrane (this work)	59.7 ± 0.5	$6.2 \cdot 10^{-7} – 1.0 \cdot 10^{-3}$	$3.2 \cdot 10^{-7}$	4 ($c = 5.0 \cdot 10^{-7} – 1.0 \cdot 10^{-4}$)	0.72	≈ 100	13 interferents (10 inorganic ions + 3 organic compounds)	peels of oranges, lemons, and bananas	104.2 – 107.6
